# Characteristics of unmanned aerial spraying systems and related spray drift: A review

**DOI:** 10.3389/fpls.2022.870956

**Published:** 2022-08-08

**Authors:** Pengchao Chen, Jean Paul Douzals, Yubin Lan, Eric Cotteux, Xavier Delpuech, Guilhem Pouxviel, Yilong Zhan

**Affiliations:** ^1^National Center for International Collaboration Research on Precision Agricultural Aviation Pesticides Spraying Technology, College of Electronic Engineering and Artificial Intelligence, South China Agricultural University, Guangzhou, China; ^2^UMR ITAP, National Research Institute for Agriculture, Food and Environment, Université de Montpellier, Montpellier, France; ^3^Institut Français de la Vigne et du vin (IFV), Montpellier, France

**Keywords:** unmanned aerial spraying systems, spray drift, downwash airflow, drift measurement, relative movement

## Abstract

Although drift is not a new issue, it deserves further attention for Unmanned Aerial Spraying Systems (UASS). The use of UASS as a spraying tool for Plant Protection Products is currently explored and applied worldwide. They boast different benefits such as reduced applicator exposure, high operating efficiency and are unconcerned by field-related constraints (ground slope, ground resistance). This review summarizes UASS characteristics, spray drift and the factors affecting UASS drift, and further research that still needs to be developed. The distinctive features of UASS comprise the existence of one or more rotors, relatively higher spraying altitude, faster-flying speed, and limited payload. This study highlights that due to most of these features, the drift of UASS may be inevitable. However, this drift could be effectively reduced by optimizing the structural layout of the rotor and spraying system, adjusting the operating parameters, and establishing a drift buffer zone. Further efforts are still necessary to better assess the drift characteristics of UASS, establish drift models from typical models, crops, and climate environments, and discuss standard methods for measuring UASS drift.

## Introduction

Unmanned Aerial Spraying Systems (UASS) consist drones that carry a spraying device. They are operated by a control system and comprise sensors to spray plant protection products. UASS have been developed rapidly during recent years as a spray tool for the application of plant protection products (He et al., 2018; Wang L. et al., 2022). According to existing reports, the use of UASS to carry out chemical spraying covers most parts of the world. In East Asia, where field conditions are limiting and where the original plant protection equipment is still in use, there is an urgent demand for UASS on the market (Lan and Chen, 2018). The number of UASS has exploded in this region. In 2014 China owned less than 1,000 plant protection drones, with an annual operating area lesser than 0.28 million ha. By the end of 2020, the number of drones in China reached 106,000, with a total yearly working area of 64 million ha (Zhang et al., 2021). In Europe, due to restrictions in application of plant protection products with aerial technology (128/CE/2009), UASS have not yet been used at a large scale yet (Reger et al., 2018). However, in mountainous grape-growing areas, producers and researchers have shown strong interest for UASS (Sarri et al., 2019; Bloise et al., 2020). The UASS can spray in the hilly and steep slope areas without being restricted by field obstacles (Delpuech et al., 2022). This has positive practical significance for separating the applicator from the tanks and replacing the backpack sprayer (Wang et al., 2020). In addition, although agricultural aviation is active on the American continent, with mainly manned fixed-wing aircraft, which are widely used in the United States, Canada, and Brazil, experimental research on UASS is also being carried out (Teske et al., 2018; Richardson et al., 2019; Li et al., 2021a,b).

UASS boast advantages in pesticide spraying. On the one hand, compared to any other ground spraying technique, the drone isolates the tank from the applicator, thus favoring operator safety (Qin et al., 2016; Morales-Rodríguez et al., 2022). As with other aerial techniques, physical damage to crops can be avoided. It can easily spray above high standing crops (bananas, corn, and rubber) and operate over complex terrain (steep slopes, terraces) where backpack sprayers are confronted to critical operator issues regarding tediousness and safety (Lan and Chen, 2018; Cavalaris et al., 2022). Moreover, exploitation costs are reduced by shortening the time of spray application and by lowering the amount of plant protection products applied (Morales-Rodríguez et al., 2022). Carbon-based fuel can also be replaced by electricity derived from renewable energies. It thus lowers the carbon impact and save costs since carbon-based fuel can be replaced by energy that, technically, could be easy to generate in a farmyard (Hussain and Nishat, 2022). Currently, UASS has been widely used over flat fields or terraces with low-lying crops, including grain crops such as wheat, corn, rice, and cash crops such as cotton, citrus, and grapes (Pan et al., 2016; Sarri et al., 2019; Wang L. et al., 2019; Chen et al., 2020a; Meng et al., 2020; Chen H. et al., 2021). Spraying with UASS has proven to be feasible in the prevention and control of crop diseases and pests by spraying insecticides or fungicides (Meng et al., 2018; Yan et al., 2022). In the case of trees grown on steep slopes, the quality of the application is partially limited by the flight altitude of the sensor and terrain following technology with the help of lidar for example (Meng et al., 2022b; Wang C. et al., 2022). Moreover, a denser crop canopy also presents limitations in terms of droplet penetration (Chen et al., 2020b; Yu et al., 2022). For these latter reasons, the application with UASS on 3D crops in mountainous and hilly areas is still being investigated.

Although the market is open to UASS, the risk of environmental drift caused by drone spray is also noteworthy (Wang et al., 2020a,^2021^). The risk of spray drift could be closely related to operational efficiency and operating parameters. On one hand, the operating efficiency of a single UASS has increased from 2 to 3 hectares per hour to the current 15–20 hectares per hour within the past 5 years (Chen H. et al., 2021). The result of single-machine efficiency implies that more chemicals can be sprayed in a short time (Wang Z. et al., 2022), however more pesticide droplets may also be scattered in the air (Liu et al., 2021). The overall environmental risks due to efficiency improvements need to be assessed. On another hand, drift can be minimized when low flying altitude is applied (1–3 m). Due to the varying growth heights of crops, the actual flying altitude is rather generally comprised between 3 and 10 m (Wang et al., 2019b,^2021^). The flight speed generally ranges between 1 and 6 m/s (Chen H. et al., 2021). Flying altitude and speed may cause the droplets to move in the air for a longer time. Nevertheless, they are also susceptible to the natural lateral wind and environmental climate, forcing which result in drift (Chen H. et al., 2021).

Studies on drone drift include theoretical (CFD simulations) and experimental studies. Current research on theoretical analysis focuses on the changes in the wind field of the UASS rotor and the movement of droplets affected by the wind field using calculations and simulations (Zhu et al., 2019; Tang et al., 2020, 2021; Zhang et al., 2022). Experimental research is mainly carried out in wind tunnels or in the field combined, with present-day climate environment and crop types. Current experimental studies on drift include the characteristics of UASS drift, drift distance, and the influence of operating parameters or spraying systems on the drift (Wang et al., 2020, 2020a; 2021). However, current research on UASS drift is still scarce. Data on the spray drift of drones and their impact on the environment are scarce, and the factors affecting drift are still being studied. Existing technical standards do not address the drift of UASS, including how to test drift in the field and wind tunnels (Wang et al., 2020). In addition to the European ban on aerial sprayers, no relevant country or region implements a specific legislation on drone drift (Reger et al., 2018).

Although drift is not a new concern, it requires further attention toward new equipment that is being widely used. This literature review focuses on the emerging issue of drift caused by UASS. Articles from scientific journals were searched and analyzed from 2014 by setting keywords, such as UAV/UASS plus spraying or drift, etc., including a part of Chinese literature indexed by the engineering index. Section “Characteristics of unmanned aerial spraying systems and spray drift” describes UASS platforms, the spraying systems and the characteristics of spray drift generated by drones. Drift evaluation protocols test methods developed for drones, and the possible environmental risks are also included. Section “Factors influencing unmanned aerial spraying systems drift” rather focuses on more fundamental processes where spraying is combined with the displacement of the UASS. This chapter reviews the factors that affect the drift of the UASS including atomization, downwash airflow, and the relative movement. The atomization factor caused by the structural design of the spraying system includes the selection of nozzles, the layout of nozzles and rotors, and the properties of the liquid (Chen P. et al., 2021). For the downwash airflow, the number and size of rotors and payload were investigated. The relative movement refers to changes in the UASS flight process that may either come from itself or from the surrounding environment, including the UASS flight parameters and natural lateral wind (Wang et al., 2020). The issue of evaporation during spraying is not considered in this article. Finally, since current research on the drift of UASS sprayers is still limited, the lack of research studies and the future research that needs to be developed are discussed in Section “Discussion and further recommendations.”

## Characteristics of unmanned aerial spraying systems and spray drift

### Characteristics of unmanned aerial spraying systems

#### Unmanned aerial spraying systems platform

Fuel-powered agricultural helicopters first appeared in Japan in the 1980s (Chen H. et al., 2021). With the recent technical developments, electrical single-rotor or multi-rotor models have gradually replaced fuel-powered helicopters (He et al., 2017; Chen H. et al., 2021). Table 1 summarizes the technical parameters of a few typical UASS. The structure of electrical rotary-wing plant protection UASS mainly comprises the rotor, tank, spraying system, control system, environmental sensor, energy system, etc. The rotor provides lift for the UASS and at the same time generates a unique downwash wind field (Zhan et al., 2022). Drone rotors available on the market are built with single rotors, two rotors, four rotors, six rotors, and eight rotors. The tank is the major element of UASS, and its volume is related to the maximum payload weight. According to Table 1, the tank volume in new models has been increasing in recent years. The initial payload range is 8–15 L, and some current models can reach up to 20–40 L. The control system and environmental sensing sensors are the fastest elements of the drone update iteration, evolving from the initial manual control mode, semi-automatic (ex. Trajectory from Point A to Point B mode) control mode to fully autonomous mode. Positioning sensors have evolved from the Global Navigation Satellite System (GNSS) with meter-level errors to Real Time Kinematic (RTK) with centimeter-level errors. In addition, air pressure sensors, ultrasonic sensors, radar, binocular vision, and other sensors used for altitude determination, distance measurement, and obstacle avoidance are constantly updated (Wang L. et al., 2019; Chen H. et al., 2021).

**TABLE 1:** The technical parameters of some typical UASS.

Model (Manufacturer, release time)	Dimensions (Frame arms unfolded, mm)	Rotors (Number*diameter *pitch, mm)	Payload (Kg)	Fully loaded weight (Kg)	Geolocation technology
T30 (DJI, 2021)	2,858 × 2,685 × 790	6*38*508	30	66.5	RTK,Horizontal ± 10 cm, vertical ± 10 cm
T16 (DJI, 2019)	2,520 × 2,212 × 720	6*33 × 177.8	16	40.7	
MG-1P (DJI, 2018)	1,460 × 1,460 × 578	4*21*177.8	10	22.5	GNSS/RTK
V40 (XAG, 2021)	2,795 × 828 × 731	2*47*457.2	16	44	RTK,Horizontal ± 10 cm, vertical ± 10 cm
P40 (XAG,2021)	2,110 × 2,127 × 555	4*40*352.1	20	45	
P20 (XAG, 2019)	1,830 × 1,822 × 452	4*33*292.1	10	28	

#### Spraying system

**FIGURE 1:**
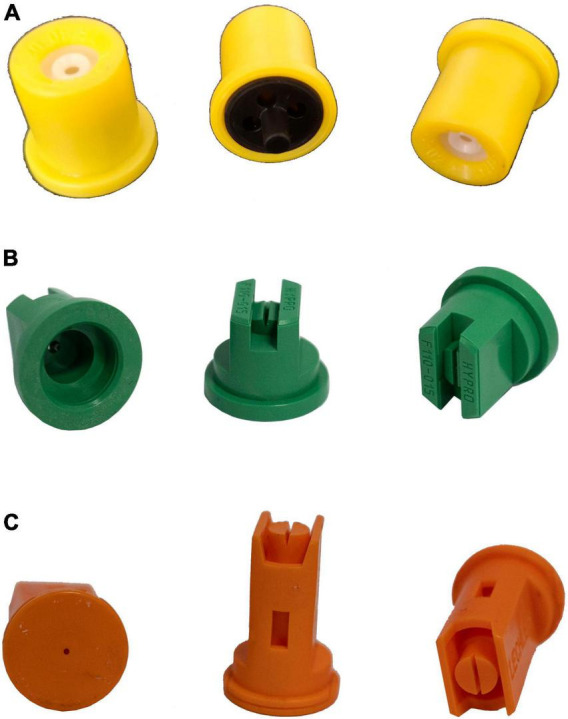
Examples of Hydraulic nozzles. **(A)** Hollow cone nozzle (TR80-02c, Lechler), **(B)** flat fan nozzle (HYPRO, 110-015), **(C)** air induction nozzle (IDK 120-01, Lechler).

**FIGURE 2:**
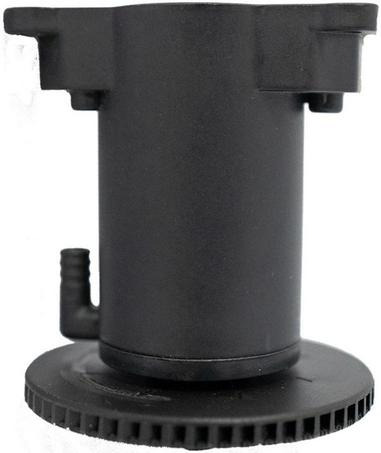
Centrifugal nozzle (2018, XAG Co., Ltd).

The nozzle represents an essential part of the UASS spraying system. As illustrated in Figures 1, 2, commonly used nozzles for UASS include hydraulic and centrifugal nozzles (He et al., 2018). Hydraulic nozzles are derived from ground spray equipment and are currently the most common type of nozzle for UASS. The chemical solution is atomized through the nozzle cavity under a given pressure and forms a liquid film. The liquid film is continuously stretched and formed into a filamentary shape under the pressure difference. When the liquid film collides with relatively static air, it splits into fine droplets (ASAE ANSI/ASABE, 2020; He et al., 2018). The hydraulic nozzle atomization can be modified by adjusting the pressure, changing the surface tension of the solution or equipping the nozzle with air inclusion or Venturi nozzles (Al Heidary et al., 2014).

The centrifugal spraying system adopted by UASS mainly consists of a rotary disc centrifugal nozzle. The rotary disc-type centrifugal nozzle comprises multiple radial grooves on the inner wall of the rotary disc (Qingqing et al., 2017). The groove ends are generally equilateral pins. The existence of radial grooves can reduce the slippage of the solution and allow the solution and rotary disc to share similar circumferential speeds (He et al., 2018). The solution in the nozzle enters the high-speed rotating turntable through the draft tube, and the droplets fly out in a spiral tangential direction along the edge of the turntable under the action of centrifugal force, forming droplets of uniform size (Gao, 2013; Qingqing et al., 2017). With a centrifugal nozzle, the spray mix relies on gravity to enter the turntable and is ejected from the radial direction under centrifugal force on radial pins (Qingqing et al., 2017). The required spray pressure is therefore, slight, resulting in a narrow droplet spectrum but also a weak droplet penetration. However, as the droplets flowing out of the nozzle do not interfere with one another, the distribution of droplet deposition is more uniform and controllable (Hayashi and Takeda, 1986). The spectrum of the atomized droplets can be adjusted by controlling the rotational speed of the spray disc in order to meet different droplet size requirements. Under the different voltages, the rotation speed of the nozzle can vary from 0 to 17,000 revolutions per minute (RPM) (Wang et al., 2020). The spray disc is not easy to clog and is particularly suitable for spraying wettable powders and suspension agents with low solubility (Qingqing et al., 2017; Wang et al., 2020). It is adapted to a high concentration of UASS chemical liquid. However, centrifugal nozzles produce fine droplets, and as their direction of movement is horizontal, the risk of drift is high (Wang et al., 2020).

In the early stages of UASS development, the flow rate could be modified by changing the nozzle type or adjusting the flight speed (Chen et al., 2020a). However, changing the nozzle implies a change in the size of the droplets. The influence of the flight speed on the droplet distribution and drift can thus be ignored. At present, the flow rate can be essentially modified by increasing the number of water pumps and nozzles and by adjusting the pump flowrate. The number of pumps and nozzles carried by drones has also been increasing as operational efficiency is being developed (Chen H. et al., 2021).

According to Table 2, the difference between both spraying systems is the range of values of the nozzle Volume Median Diameter (VMD). For the hydraulic spraying system, the droplet size is affected by the nozzle type, operating pressure, and the nature of the solution. For centrifugal nozzles, the significant factor is the speed of the spray plate. The droplet size is strongly related to drift (Al Heidary et al., 2014). Choosing a nozzle with a larger VMD can reduce the risk of drifting in the spraying system, such as air induction nozzles that are widely used in boom sprayers. However, choosing anti-drift nozzles on UASS may not always be suitable for crop protection. Due to load limitation, the improvement of the spraying quality of UASS implies a reduction in the atomized particle size in order to ensure a higher droplet density and coverage. However, by reducing the size of the droplets, the risk of drift is increased. For UASS, improving the spray quality and reducing the risk of drift have contradictory effects.

**TABLE 2:** Characteristics and comparison of different UASS spraying systems.

Spraying system	Nozzles	VMD (μm)	Droplet size adjustment method
Hydraulic spraying system	Flat fan	110–200	Adjust pressure, solution properties, nozzle type
	Hollow cone	90–150	
	Air induction	220–400	
Centrifugal spraying system	Centrifugal	90–300	Change the speed of the spray plate

### Characteristics of unmanned aerial spraying systems spray drift

#### Downwash and outside airflow

The most significant feature of rotary-wing UASS is to carry one or more rotors (Li J. et al., 2018). However, rotor movement can also cause vortex or turbulence (Fengbo et al., 2017; Wang et al., 2020). When the wing generates a positive lift due to the pressure difference between the upper and lower wing surfaces, the high-pressure airflow below follows the wingtips, then rolls upwards and flows toward the lower pressure upper side of the wing, forming a spiral-shaped vortex (Wen et al., 2018). Wingtip vortices are not unique to drones, and they can also occur in helicopters and fixed-wing aircraft (Mickle, 1996). However, the high-speed rotor of the drone will cause the movement of the droplets under the rotor to be more complex. With a stronger rotor downwash, the vortex in flight is stronger (Zhan et al., 2022). Under the entrapment of the vortex, a greater number of droplets spread to both sides of the route, further worsening the downwind drift (Wang et al., 2020a). This vortex generated by the joint action of the rotor downwash airflow and the outside air is a major factor affecting the drift of UASS spray (Tang et al., 2021). Two types of outside airflow exist: the relative air movement caused by the drone’s forward speed and the natural wind. Wen et al. (2018) showed that a spiral wake occurs behind the aircraft when the flight speed exceeds 3 m/s. The higher the speed, the longer the spiral vortex prevails in the air. Moreover, when the drone hovers, instead of drifting, the droplets fall directly to the ground with the downwash of the main rotor (Wen et al., 2018). Results concerning hovering situations are derived from software simulations, therefore, the same observations might not be made in the case of field trials. On one hand, even when no environmental wind blows, fine droplets sprayed by UASS with centrifugal nozzles can drift beyond 4 m downwind due to the effect of the rotor wind and the Brownian motion (Wang et al., 2020). On the other hand, since the UASS operate above the canopy droplets in the air can easily drift outside the crop with a crosswind (Li L. et al., 2018). Consequently, UASS drift cannot be totally avoided under the combined effects of the rotor wind field, natural wind, and sprayer movement.

#### Unmanned aerial spraying systems drift measurement method

Drone drifting still lacks a standard testing method, and existing research mainly refers to the ISO22866 standard (Iso, 2005). The drift phenomenon can be evaluated through sedimentation and/or airborne drift according to the spatial position of collectors (Grella et al., 2017). Sedimentation drift involves the collection of ground deposition at different distances downwind that is typically used to assess water course exposure (Wang J. et al., 2018). Airborne drift consists of the collection of droplets during their transport in the atmosphere typically at several meters from the field edge and at different heights reaching several meters above the ground (Wang et al., 2021). This airborne drift can be used to evaluate the transport of droplets and further consequences in terms of resident exposure (Al Heidary et al., 2014).

In the existing literature, UASS drift tests are mainly carried out in the field (Wang et al., 2019a,^2020^, ^2021^). Table 3 summarizes the test methods from certain field tests found in the literature. The drift collection is made in the downwind direction and perpendicular to the UASS flight direction (Wang et al., 2020). For the different spatial positions of the collectors, spray drift is detected by extracting a dye tracer from the polyethylene wire, active sampler or rotary impactors for catching airborne drift. Petri dishes, Mylar cards or filter papers are used as collectors to sample sedimentation drift (Wang et al., 2019a,^2021^; Ahmad et al., 2022). According to the statistics in Table 3 provided by the literature, the sampling points of sediment drift are usually arranged in non-target areas ranging from 1 to 50 m, while airborne drift includes one or more sampling points within 50 m.

**TABLE 3:** Field test methods for UASS spray drift evaluation in the literature.

UASS sprayer	Fluorescence tracer	Testing method (Sampling location)	Material	References
Z-3	Rhodamine-B	Sediment (2–100 m) and Airborne (2, 50 m)	polyester card (ϕ = 90 mm) and polyester fiber (ϕ = 1 mm)	Xinyu et al., 2014
Yamaha R-MAX II	/	Sediment (7.5–48 m) and Airborne	Deposition sheet (40 * 25 cm) and SKC AirCheck HV30 sample pump	Brown and Giles, 2018
3WQF120-12	Brillant sulfoflavin dye (BSF)	Sediment (1–20 m) and Airborne (5,10,20 m)	Petri dishes and rotary impactors	Wang X. et al., 2018
3WQF80-10	BSF	Airborne drift	A cuboid aluminum sampling frame (5 m × 5 m × 2 m)	Wang X. et al., 2018
X-4	Tartrazine solution	Sediment and Airborne (5,10 m)	filter paper and water sensitive paper	Li J. et al., 2018
3QF120-12	Rhodamine-B	Sediment (1–50 m) and Airborne (10,25,50 m)	mylar card (10 × 8 cm), monofilament line (Ø = 0.45 mm)	Wang X. et al., 2018
MG-1S	Allure red	Sediment drift (0.5–12.5 m)	Mylar cards	Chen et al., 2020a,b; Chen S. et al., 2020
P20 (XAG)	Rhodamine-B	Sediment (2–50 m) and Airborne (2,12 m)	mylar plate (5 × 8 cm^2^) and monofilament line (φ = 0.6 mm)	Wang et al., 2020
3WQF120-12, 3WM6E-10, 3WM8A-20	Pyranine	Sediment (2 m) and Airborne (2–20 m)	Petri dishes, rectangle collection frames with polyethylene tubes (5.5 × 2.0 m), rotary samplers	Wang et al., 2021

The He research team proposed a 3D mass balance test method consisting of a 5 m × 5 m × 2 m tunnel frame with ∅2 mm drift collection lines on four sides (left–right–ground–top) to collect the droplets sprayed inside the tunnel by a UASS () (Wang et al., 2016). Quantitative information can thus be obtained along the four directions, although information is lacking at different distances on the ground. Wang et al. (2021) used a near-ground drift test platform with Petri dishes to collect sedimented droplets at different distances downwind from the UASS route. Wang et al. (2019a) and Wang et al. (2020) arranged the collection poles at a height of 1 m within a 2–50 m range in the downwind direction and fixed Mylar plates (5 × 8 cm) to each collection pole. The airborne drift near the ground was estimated after recovering the Mylar plates. Assessing sedimentation drift is the most common method in spray drift research, and it reflects the real value of ground drift at different distances from the downwind direction. However, data on the vertical spatial distribution of drift is still lacking. In order to efficiently understand the spatial distribution of droplets on the downwind side of a UASS flight path, both sediment and airborne drift need to be considered.

**FIGURE 3:**
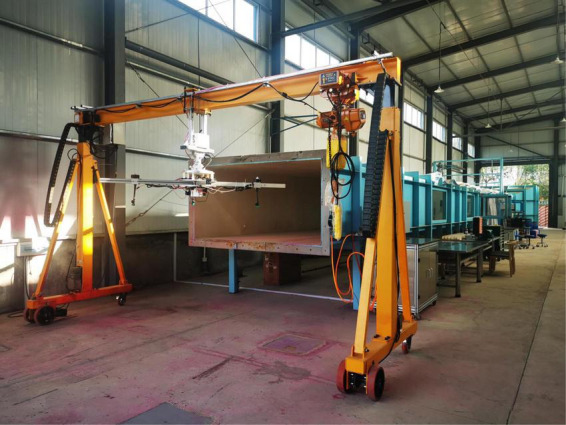
The UASS spraying test bench in South China Agricultural University.

Since field tests can be easily affected by weather conditions, wind tunnels are a solution to provide stable and controllable wind conditions, allowing for repeatable operations (Iso International Standard, 2009). Wang et al. (2020b) placed the single rotor and nozzle of the drone in a wind tunnel. The rotor refers to one single spray unit of a quadrotor UASS “3WQFTX-10” (Anyang Quanfeng Aviation Plant Protection Technology Co., Ltd., China), with a size of 76.2 cm. Ling et al. (2018) placed a UASS carrying a spraying system inside a 2 m × 2 m wind tunnel for spray testing. The UASS model used here was a miniature version. Although these studies attempted to test the UASS in a wind tunnel, the use of a single rotor or the reduction in the size of the UASS may differ from reality. A research team from South China Agricultural University and Nanjing Research Institute for Agricultural Mechanization, China, built a set of UASS test platforms (as illustrated in Figure 3). The test platform can hold up to 4, 6, and 8 rotors (adjusted as needed). The rotor speed can be adjusted within the range of 600–2,500 RPM. The spraying system is located under the rotor, and can be installed with a hydraulic spraying system or a centrifugal spraying system, where the position of the nozzle relative to the rotor can be adjusted freely. In addition, the test platform can adjust the pitch angle from –30^°^ to 30^°^. Liu et al. (2021) combined the UASS platform with the wind tunnel and placed the platform at the extremity of the wind tunnel in order to build an indoor drift test device. Although the sampling area is not located inside the wind tunnel, this method is a good attempt to reduce disturbances from natural environmental conditions.

#### Potential environmental risks

The UASS uses low application volume rates for spraying because of the limited payload (Zhan et al., 2022). Compared with ground sprayers, the amount of spray per unit area of drones is less even though the rate of active substance can be equivalent (Qin et al., 2016; Wang G. et al., 2019). The drift rate (as normalized by the application volume, ISO 22866) is therefore not significantly reduced. The conclusions of the study by Wang G. et al. (2019) are that pesticide droplets from multi-rotor drones drift further away than with a traditional backpack sprayer. In addition, the amount of drift in the air is greater (Wang G. et al., 2019). Indeed, according to a study by Li L. et al. (2018) the multiple rows of vertical crop canopies can effectively prevent droplets from moving during ground equipment spraying, thus resulting in a lower extent of drift outside the crop than with UASS. The Wang field experiment study also demonstrated that the UASS drift of almost all treatments at 50 m was lower than the detection limits, and that the drift distance of the UASS model was much shorter than that of an aerial manned aircraft sprayer (Wang et al., 2020). However, the above conclusions are particular cases that depend on the spraying system, crop type and operation scenario.

Xu et al. (2020) performed preliminary research on applicator exposure in a rice paddy by multi-rotor UASS. They clearly highlighted that the risk of exposure using UASS applicators was almost zero due to the separation between the applicator and application machine. In contrast, backpack sprayer application resulted in entire body exposure of the applicator to the pesticide. Yan et al. (2021) compared the amount of insecticide droplet drift with the mortality of bees for multi-rotor plant protection UASS and for electric backpack sprayers. After pesticide application by the multi-rotor drone and electric backpack sprayer, the droplet deposition at a distance of 5 m downwind was 0.107 9 μg cm^–2^ and 0.002 2 μg cm^–2^ respectively. The number of bee deaths caused by the plant protection drone application drift was 62.9 fold that of the electric backpack sprayer (Yan et al., 2021). Current UASS drift research focuses on sediment and airborne drift, while the impact on non-target organisms is still limited. Further tests are still necessary to evaluate the environmental risks of drone drift.

## Factors influencing unmanned aerial spraying systems drift

### Atomization and sprays

#### Nozzles

The nozzle is at the core of the spraying system as it plays a key role in spray atomization. Spray atomization refers to the process of spraying a liquid into a gas medium at high speed through a nozzle, dispersing and fragmenting it, and finally forming fine particle droplets (He et al., 2018). Both the size of the droplets generated by atomization and the proportion of fine droplets have an impact on the drift (Al Heidary et al., 2014). In the spraying process of ground spray equipment, air induction fan nozzles are used in specific anti-drift scenarios. Table 4 summarizes drift test results from UASS equipped with different nozzles in the field. Regardless of the different UASS models and test areas, IDK 120-015 presents a better anti-drift effect than TR 80-0067. Hollow cone nozzles produce finer droplets and are often used for pest control in orchards; IDK nozzles produce larger droplet sizes than flat fan nozzles. The average VMD (DV50) values of IDK 120-015 and TR 80-0067 in this test were 114.9 and 312.6 μm, respectively, and the proportions of droplets with a particle size smaller than 75 μm were 16.1 and 1.8%, respectively. The air induction nozzle can produce coarser droplets, thus reducing the risk of droplet drifting (Wang et al., 2020a).

**TABLE 4:** Comparison of the 90% drift distance with different nozzles and UASS in the literature.

Nozzles	UASS	Dv50/μm	Wind speed (m/s)	Distance 90% of total sedimentary drift (m)	References
Centrifugal nozzle (XAG company)	P20 (4-Rotor)	100	1.16 ± 0.06	13.2	Wang et al., 2020
		150	1.30 ± 0.05	12.0	
		200	0.61 ± 0.03	5.7	
Hollow cone nozzle, TR 80-0067	3WQF120-12 (Helicopter)	114.9 ± 0.7	3.31 ± 0.17	9.99	Wang et al., 2021
	3WM6E-10 (6-Rotor)		3.79 ± 0.58	11.53	
	3WM8A-20 (6-Rotor)		3.47 ± 0.37	11.70	
Air-injector nozzle, IDK 120-015	3WQF120-12 (Helicopter)	312.6 ± 1.8	3.11 ± 0.40	9.13	
	3WM6E-10 (6-Rotor)		3.45 ± 0.46	7.90	
	3WM8A-20 (6-Rotor)		3.37 ± 0.56	13.62	
Flat fan nozzle, LU 120-02	3WQF120-12	268.6	2.82 ± 0.76	10.05	Wang J. et al., 2018

A correct selection of nozzles has significant effects in reducing drift (Herbst et al., 2020; Wang et al., 2020). According to Table 4 the result of 90% of total sedimentary drift locations correlates strongly with droplet size (Dv50). The influence of the nozzle on drift depends on the droplet size (Dv50) produced by atomization. The larger the droplet size, the better anti-drift performance (Wang et al., 2020). Larger droplets, which hardly moved upwards with the vortex, traveled much shorter distances and floated at lower altitudes. When the size of the droplets increased, their maximum drifting distance gradually decreased and was less affected by crosswind speed and direction (Wang J. et al., 2018; Wang et al., 2020, 2021). This conclusion has been verified in several of the studies presented in Table 4. When the crosswind blew from the right-hand side, large droplets (200 and 400 μm) tended to deposit faster and closer to the swath, while fine droplets (50 and 100 μm) were displaced by the crosswind with a strong non-uniform spatial distribution and a tendency to float toward the far left-hand side (Tang et al., 2021). The drift distance of droplets gradually decreases as the droplet size increases. Research by Wang et al. (2020) shows that large droplets are more affected by gravity and mainly deposit on the lower half of the 2 m, while fine droplets remain suspended in the air and are less affected by gravity, thus leading to a higher slope of airborne drift at 12 m.

#### Layout of nozzles

**FIGURE 4:**
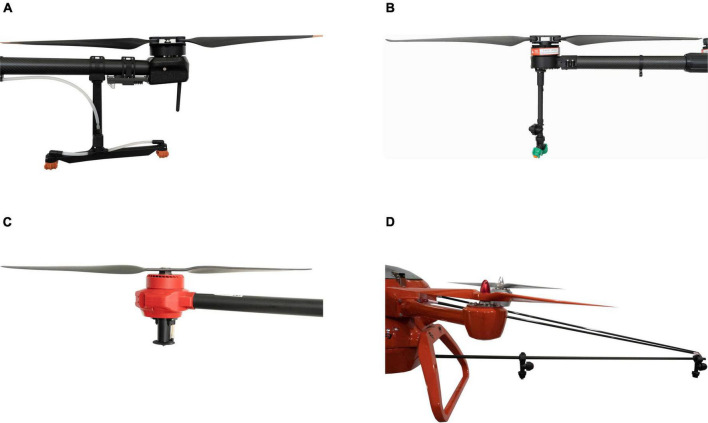
The relative position of the rotor and the nozzle. **(A)** Inside under the rotor (T30, from DJI), **(B)** below the rotor (extended, 3WWDZ-16, from Tuogong), **(C)** below the rotor (P30, from XAG), **(D)** boom (kongzhongbaoma, from SCAU).

The location of the nozzle under the rotor affects the movement of the droplets (Chen H. et al., 2021). As illustrated in the Figure 4, four standard layouts of rotors and nozzles are possible. (i) The nozzle can be located directly below the rotor, (ii) the nozzle can be located directly below the rotor (extended), (iii) the nozzle can be located inside the rotor, or (iv) the nozzle can be separated from the rotor (spray boom). However, studies on the impact of the spatial layout of rotors and nozzles on spray drift are still scarce. The typical nozzle arrangements are spray boom and vertical suspension under the rotor. The sensitivity to spray drift depends on the position of the nozzle. Indeed, the nozzles at the two extremities of the boom are sensitive to the rotor vortex. The closer the nozzle to the wingtip of the rotor, the greater the amount of droplets drawn by the wingtip vortex (Wang J. et al., 2017). To reduce spray drift, the length of the boom (similar to Figure 4D) should not be greater than the diameter of the rotor (Chen H. et al., 2021) as has been advised for larger aerial spraying systems. A reduction in the distance between nozzles can also decrease the droplet drift caused by wingtip vortices (Wen et al., 2018).

#### Adjuvant and formulation

Adjuvant can significantly reduce the surface tension of the solution (Meng et al., 2021, 2022a). In a field trial study, Silwett DRS-60, ASFA + B, T1602, Break-thru Vibrant, QF-LY and Tmax could reduce spray drift by 65, 62, 59, 46, 42, and 19%, respectively, in comparison with water. The adequate concentration of adjuvants can reduce the percentage of fine droplets and thus significantly decrease the risk of drift in agricultural spraying (Wang X. et al., 2018). Wind tunnel experiments in different meteorological condition also demonstrated that the addition of spray adjuvants to the spray solution can affect the level of spray drift level (Wang et al., 2020a). The effect of adjuvant has also been found to lessen drift by modifying the surface tension of the solution, thus contributing to a reduction of the proportion of fine droplets. It therefore plays a significant role in reducing the drift risk of UASS.

Ultra-low volume spraying by UASS entails exigent demands in pesticide formulations. The drift of herbicides generally produces a higher impact on the environment than for fungicides and insecticides. In the early stage of UASS application, the blind use of herbicides to affect non-target organisms is a common strategy (Shan et al., 2021). While Japan developed drone sprayers earlier, herbicides were processed in the form of granules specifically for drone application according to the properties of drone aircraft spraying (Yuan et al., 2018). Granules can be employed in paddy fields such as rice, thus reducing environmental risks for non-target areas. Further efforts in the future will still be necessary to develop adequate pesticide formulations for UASS (Yuan et al., 2018).

### Downwash airflow

Rotor airflow is a typical feature in UASS (Zhan et al., 2022). The airflow of the rotor directly affects the movement of the droplets in space. It is the main factor that affects the airborne delivery of droplets to the target but also the leading cause of drift (Li J. et al., 2018). The following section summarizes the factors that cause variations in rotor airflow, including rotor and payload.

#### Rotors

**FIGURE 5:**
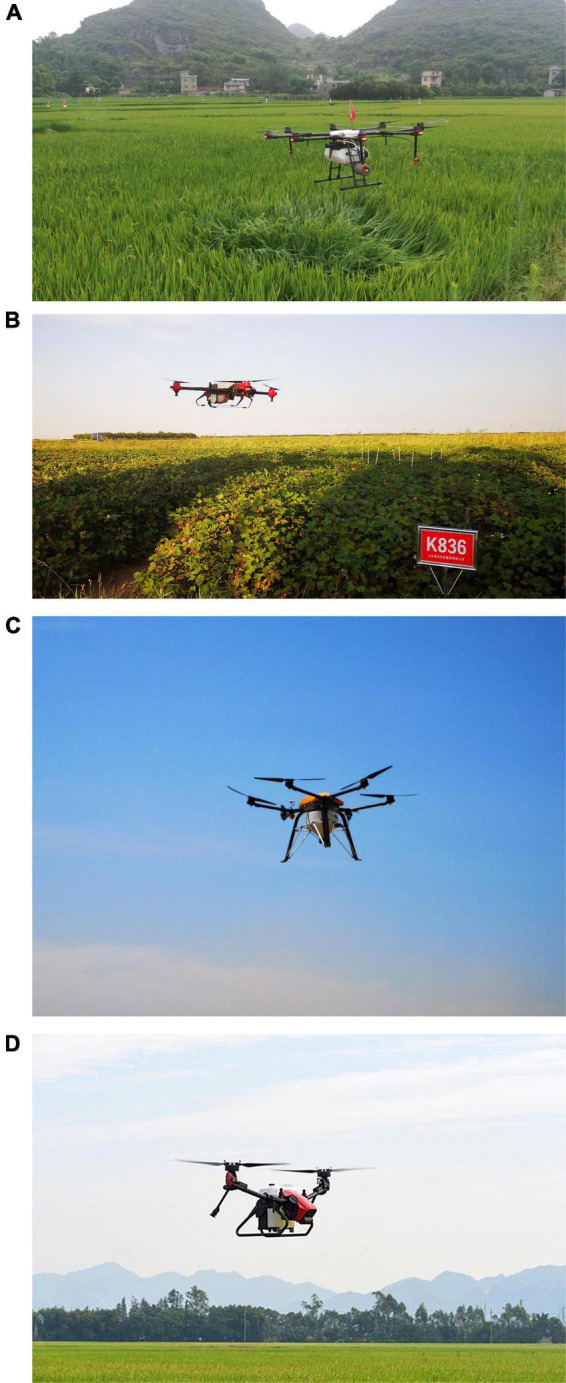
The UASS with different numbers of rotors. **(A)** Eight-rotor UASS (MG-1P, from DJI), **(B)** quadrotor UASS (P30, from XAG), **(C)** six-rotor UASS (M45, from GKXN,China), **(D)** two-rotor UASS (V40, from XAG).

The UASS are divided into single rotor and multi-rotor Systems. Figure 5 introduces several multirotor UASS and Table 5 summarizes the effective coverage area and average wind pressure of certain UASS. A single-rotor therefore covers a larger effective area than a multi-rotor. However, the take-off weight of the multi-rotor is not lower than that of the single-rotor. In terms of the downward wind pressure generated by the rotor, the multi-rotor performs better than the single-rotor; however its effect on drift cannot yet be explained.

**TABLE 5:** Comparison of effective wind field area and average wind pressure of some UASS.

Type	Model	Rotor diameter (mm) * number	Cover effective area^a^/m^2^	Standard takeoff weight/kg	Average wind pressure^b^/(kg⋅m^–2^)
Oil single rotor	3WQF120-12	2,410 * 1	4.56	42	9.21
Electric single rotor	S40-A	2,400 * 1	4.52	40	8.85
Electric multi-rotor	V40 2021	1,194 * 2	2.24	44	19.64
	P40 2021	1,016 * 4	3.24	45	13.89
	P80 2021	1,194 * 4	4.48	80	17.86
	T16	838.2 * 6	3.3	41	13.67
	T30	965.2 * 6	4.38	66.5	15.18

^a^The cover effective area is equal to the coverage area of the rotor multiplied by the number of rotors.

^b^The average wind pressure is equal to the take-off weight divided by the effective coverage area.

Even though data on the way the type and number of rotors affect drift is still lacking, the influence of rotors on drift has been acknowledged (Richardson et al., 2019). As mentioned in Table 4, Wang studied the drift characteristics of hollow cone and air-injector nozzles mounted on UASS with different numbers of rotors (Wang et al., 2021). Based on 90% of the total drift distance, they demonstrated that the single rotor case always provided lowest drift distances. Following a computer simulation, Tang et al. (2021) observed that the largest droplets (200 and 400 μm) would be deposited near the swath, while the smallest droplets (50 and 100 μm) would remain airborne on the far left-hand side. Since the application height of the helicopter was low, a spanwise vortex appeared near the ground on the left-hand side of the helicopter. As a result, fine droplets were lifted due to the strong downwash flow while larger droplets were deposited before entering the vortex (Tang et al., 2021). These findings could be further exploited in order to significantly reduce the spray drift. In the case where a stronger downwash airflow would be produced, the effect of the vortex would be more prominent, and a greater amount of droplets would drift toward both sides of the route owing to the vortex wake of the UASS sprayer (Wang et al., 2021). Focusing on droplet deposition, the droplets were concentrated on 19.37% of the surface without a downwash flow field. The deposition area was a regular rectangle with a width of 2.6 m, which is the target area. When a downwash flow field was activated, the drift distance of the droplets increased and a greater amount of droplets traveled to non-target areas. The width of the droplet deposition area was 12.8 m, and droplets were observed on 41.06% of the test area (Shi et al., 2019). Furthermore, it is generally believed that apart from favoring the displacement and deposition of droplets, the external high-speed airflow would also lead to a second atomization, resulting in a larger variation in droplet sizes (Butler Ellis et al., 2002; Ferguson et al., 2015; Wang et al., 2021).

#### Payload

The tank represents the most significant constituent of airborne equipment as it defines the payload and productivity. Its shape and size affect the UASS weight and control performance of the entire body and can even affect the distribution of the downwash airflow (Li J. et al., 2018). The spray method found on UASS is an air assisted spraying system similar to that of an orchard sprayer. Airspeed and air volume are the main factors in orchard spray technology that affect the distribution of deposits inside and outside the fruit tree canopy (Zhai et al., 2018). The airspeed and air volume of the orchard sprayer are obtained by adjusting the speed of the fan (Balsari et al., 2019). The difference between the UASS and the orchard sprayer is that the wind speed and air volume produced by the orchard sprayer are stable and controllable. The wind field generated by the UASS rotor is affected by external factors and is relatively uncontrollable. Indeed, the lift generated by the UASS rotor(s) is related to the load that is constantly changing because of the continuous discharge of the tank mixture (Zhan et al., 2022). According to a study, the RPM of each rotor blade was found to decrease by 14–20% as the payload decreased from 10 to 0 kg (Ismail et al., 2021). Therefore, in present-day spraying activities, changes in RPM could produce a downwash airflow pattern that constantly varies from the starting point up to the finishing point. This could have an effect on the distribution of the pesticide along the flight pathway (Ismail et al., 2021).

At present, 90% of electric multi-rotor drones have a tank capacity smaller than 15 L (Wang et al., 2020), but the developmental trend is now to increase the load. Some drone companies have even released electric drones with 40 L payloads, such as DJI’s T40 and XAG’s P40. The load factor (the ratio of load/total weight) has become an essential factor that can change wind strength of the rotor. As the weight of the UASS decreases, the downwash decreases, thus reducing its ability to draw droplets toward the ground and thereby further increasing airborne drift (Teske et al., 2018). The extent to which the payload affects drift still needs to be further investigated with subsequent research.

### Relative movement

The drone constantly moves during the spraying process. Due to its displacement or to variations in external factors, the airflow can change (Wang L. et al., 2019; Tang et al., 2020). First of all, the forward movement of the UASS itself involves flight parameters, including flight speed, flight height, and flight direction. Secondly, variations in the external natural environment take place, such as natural wind blowing in the field. This section summarizes and discusses the factors related to relative motion and potential effects on spray drift.

#### Flying speed

While the rotary wing drone hovers, the wingtip vortex flows outwards to the sides of the fuselage (Wen et al., 2018). However, as the drone moves forward, a spiral wake vortex develops behind the fuselage (Wen et al., 2019). The greater the flight speed and the higher the flight altitude, the farther the diffusion distance of the wake vortex. Wen et al. (2018) studied a single-rotor UASS in a CFD simulation analysis. Results indicated that 38% of droplets drifting in the air were due to the spiral wake vortex when the flight speed was 5 m s-1, the flight altitude was 3 m, and the particle size was less than 100 μm. The 100 μm droplets account for about 80% of the total number of drifting droplets (Wen et al., 2018; Wang et al., 2020). In the case when the drone flew too fast (more than 5 m s-1), the direction of the downwash airstream of the rotor changed from vertical downward to obliquely downward due to the relative moving external wind, which weakened the pressure effect on sprayed droplets. The horizontal velocity component of the downwash airflow contributed to an increase in the external wind speed flowing opposite to the flight direction, and thus aggravating the spray drift toward the rear of the fuselage (Wang et al., 2020). Consequently, the flight speed was found to produce a significant effect on spray drift characteristics for UASS aerial application indeed, a reduction in flight speed could effectively decrease the potential spray drift (Teske et al., 2018; Wang et al., 2020, 2020a; Zhang H. et al., 2020).

#### Flight altitude

Airflow control has been achieved in orchard spraying by adjusting the distance between the nozzle and the spray target (Balsari et al., 2019). Generally, a reduction in the distance from the target should ensure sufficient air volume and air speed, while simultaneously decreasing the drift during spraying. The flight altitude refers to the height of the drone relative to the crop, which is the shortest distance the droplets need to travel to reach the surface of the target. Changes in altitude ought to affect the strength of the rotor wind field (Wen et al., 2018; Zhang H. et al., 2020). Indeed, the higher the altitude, the weaker the downwash airflow of the rotor at the top of canopy, and more easily sprayed droplets can drift with the crosswind (Wang et al., 2019a). Wang used a QuanFeng120 UASS in a pineapple field under various meteorological conditions. When the operation altitude was less than 2.5 m, the mean speed varied between 1.14 and 2.82 m/s, and the 90% spray drift distance remained within a 10 m range (Wang J. et al., 2018). Considering an operation altitude up to 3.5 m and the natural wind speed ranging between 2.02 and 3.59 m/s, the 90% spray drift distance can reach 33.54–46.50 m (Wang J. et al., 2018). Various experimental studies all come to the same conclusion that the maximum flight altitude should not be above 2.5 m in order to reduce the extent of droplet drifting (Wang J. et al., 2018).

#### Flight direction

Two concepts of flight direction are investigated here. The first concerns the forward and backward movement of the aircraft during route planning. Wang C. et al. (2018) studied the influence of forward and backward motion on droplet deposition in a tunnel frame of 5 m × 5 m × 2 m with 2 mm diameter drift collection lines on four sides (left—right—ground–top). When the UASS flew forward, the deposition rate ratio of downwind varied between 57.3 and 64.8%, while the bottom part varied between 30.3 and 38.8%. However, the deposition rate ratio of downwind decreased to 24.4–28.7% when flying backward, and the bottom part increased to 51.5–60.4%. As a result, the deposition rate of the bottom part of backward flight can reach 60% in comparison with forward flight. Therefore, the backward direction had a better result and allowed for a reduction in drift, optimizing the deposition rate on the target plant and the utilization of pesticides (Wang C. et al., 2018). However, this result was caused by the asymmetric structure of the single rotor UASS, and may not be applicable to the symmetrical multi-rotor UASS.

The second concept is the movement (perpendicular or parallel) of the flight route relative to the row of crops. When an application operation proceeds in a perpendicular direction to the row orientation, a higher proportion of drift can be observed in comparison with an application operation that runs parallel to the row orientation. This is attributed to the high proportion (> 50%) of gaps in the canopy parallel to the wind direction (Brown and Giles, 2018). Consequently, a UASS flying backward or parallel to the direction of a row of vines should significantly reduce the risk of drift. However, these research data remain very specific, and such a conclusion may vary according to the different UASS models and crop types.

#### Crosswind

The main feature of rotary-wing UASS is airflow, and often natural wind interferes directly with the airflow distribution of the rotor during operation (Li J. et al., 2018). Similarly to any other type of spraying system, the influence of crosswind on the drift of UASS remains significant (Wang C. et al., 2018). Studies have demonstrated how, under conditions of average temperature of 31.5^°^C and average relative humidity of 34.1%, the effect of crosswind can be more significant than the flight height and flight speed of the UASS (Wang X. et al., 2017). Consistently with the effect of flight altitude, crosswinds tend to reduce the strength of the vertical downward rotor wind field, thus causing droplets to deposit along the downwind side, reducing the amount of deposition in the target area and increasing the proportion of drift (Wang X. et al., 2018).

## Discussion and further recommendations

### Optimization of spraying system and structure layout

At present, the Chinese market alone is concerned by more than 178 types of agricultural drones, and the spraying systems carried by the drones are also very diverse (He, 2018). The spraying systems proposed by manufacturers can differ significantly, and models from a single manufacturer but produced at different periods can also be different. However, these drones dating from different periods are widespread in the market. Due to this fact, a universal operating rule or anti-drift suggestion is difficult to establish. Therefore, the UASS system structure design still needs to be further improved corresponding technical standards need to be set. Drone manufacturers have focused on improving the drone platform during the previous development processes, such as positioning accuracy, autonomous control, and environmental sensing devices. However, the spraying system, as a core component of the drone, has been ignored (Li J. et al., 2018). Spraying studies are scarcely conducted before drone manufacturers release drones, while they are more frequently based on existing drones for testing. In subsequent developments, an upgrade of the drone spraying system could become a primary solution.

The choice of spray head type should take into account the application scene, the spray purpose, and its chemical formulation. It is recommended that target crops and environmental conditions be included, but this rather relies on experimental data. The centrifugal nozzle presents certain advantages in terms of droplet size, controllability and a reduced relative span (Qingqing et al., 2017). However, most of the droplet classification of current centrifugal spraying systems lies within the fine particle range. The reduction in the drift of the centrifugal spraying system using chemicals and adjuvant is also an issue that deserves improvement. In addition, the simple choice of hydraulic anti-drift nozzles to reduce drift may lead to reduced spray coverage. Therefore, it is necessary to equilibrate the relationship between drift and deposition distribution for hydraulic nozzles. The selection of formulations may require a focus on the risks related to herbicides. Drones carrying rice seeds and fertilizer granules have appeared and are employed in China (Song et al., 2018). The spreading of herbicide particles using drone based spreading devices may become a novel direction of research.

Optimization of the layout of nozzles and rotors is significant factor in reducing drift (Wen et al., 2018). The characteristics of the nozzle vary with the requirements of the application scenario (Chen et al., 2020a). A combination between the characteristics of the rotor wind field and of the nozzle spray should be made in order to select the most optimal spatial layout. This would be the most crucial and effective solution to solve the issues in spray drifting. The selection of the appropriate nozzle according to the rotor downwash flow field, although a critical issue for UASS, has not been sufficiently described in the existing literature. On one hand, the number of rotors and downwash flow field intensity is strictly an engineering issue related to the design, stability and payload capacity of UASS (Zhang H. et al., 2020). Since rotors are conveniently employed to support nozzles, the downwash field flow may contribute toward droplet penetration into the foliage (Zhang S. et al., 2020). On the other hand, the choice of the nozzle is depends on agronomical specifications (expected dosage/ha, spray quality, as well as technical possibilities in terms of nozzle flowrate, nozzle technology, etc. Such a pragmatic approach leads to a few practical consequences in terms of spray deposition and spray drift which are well described in the literature. The physical description of the rotor and nozzle combination is possible in a fixed position but may become more complex when considering the travel speed and influence of atmospheric conditions. In this sense, the effective horizontal spray distribution (spray swath) for UASS is not easily predictable and still needs to be experimentally investigated. In addition, the load in the tank, which constantly decreases during the spraying flight, tends to affect the rotor thrust. Attention should therefore be paid to the manner in which the rotor wind field variations can affect the spray quality and drift.

### Drift test database and modeling

The development of drone spray technology is relatively new, and the UASS spray drift data base is still limited (Wang et al., 2020, 2020b). According to the study developed in Part 3, the drift of UASS is simultaneously affected by multiple operating parameters. Current research focuses on the influence of a specific factor or a small number of factors on drift while ignoring the interactions between multiple factors (Chen P. et al., 2021). The choice of operating parameters can be easily affected by the experience level of the operator, and the use of models can reduce the risk of drift caused by human decision-making errors (Chen et al., 2022). The selection of operating parameters for drone sprayers relies on a large number of field trials. The conduction of further assessments on larger-scale application fields would help to optimize operating parameters and fully understand the potential and limitations of UASS spray technology. According to recent observations, future research should incorporate more parameters, including system parameters, operating parameters, and environmental parameters, into the scope of the study so as to build a more accurate spray model. The existing AGDISP (Agricultural Dispersal) and CHARM (Comprehensive Hierarchical Aeromechanics Rotorcraft Model) were not originally designed for UASS sprayers. The previous models could not include all UASS platforms, rotor configurations, and spraying system types. However they could still act as a reference for UASS drift models (Chen H. et al., 2021). Therefore, a large accumulation of simulation or field data can provide the opportunity to establish a suitable model for UASS. Nevertheless, as the weather and crop structure in field experiments remain uncontrollable variables, it is challenging for these variables to be integrated into a decisive strategy (Bartzanas et al., 2013). Numerical modeling techniques such as CFD can effectively quantify the impact of mechanical designs, environmental parameters and weather conditions in a virtual environment. In addition to the drone itself, wind conditions and crop canopy are also important influencing factors. Hong et al. (2021) proposes a review of fluid dynamic approaches of spray drift taking into account influencing factors (including droplet size, wind conditions, and canopy interaction) into account to build accurate spray models, however this review does not concern the specific case fo UASS. In the future, a greater number of CFD studies will be implemented for the range of conditions for evaluating multi-rotor UASS to be expanded, thereby forming a modeling method to optimize UASS performance systematically.

In summary, the drift of UASS is an inevitable phenomenon. However, the establishment of reasonable measures, such as suggesting drift buffers throughout test data or models, is a necessary step toward drift reduction. Test data can help optimize the model in order to guide the process of selecting operating parameters. In addition, the structural design of the UASS sprayer system is still at a stage of continuous improvements, while the accumulation of test data should contribute to further improve the system.

### Standardization of measurement methods

Table 3 in section “Unmanned aerial spraying systems drift measurement method” highlights a lack of consistent test protocols in existing research projects. Many UASS spraying systems are available, and the types of sampling collectors and collection locations are also diverse. Therefore, a summary and comparison of existing research data are difficult to make. The ISO22866 standard provides a field drift test method, which can be used to compare the drift characteristics and environmental risks between different types of spray equipment. However, it may not be suitable for the UASS drift test. For example, it is necessary to determine a unified test method according to various typologies of UASS, including a number of spraying systems and spatial layouts. Including the upwind drift data caused by the UASS wingtip vortex into the scope of the evaluation is a necessary step. NY/T 3213 is China’s first agricultural UASS industry standard (Zhang S. et al., 2020). The standard determines the modeling rules, quality requirements, inspection methods, and rules of the UASS. However, the standard only defines the measurement of the UASS spray width, and the UASS drift test method is not mentioned. In order to further clarify the drift characteristics of UASS and to establish a drift model or database of UASS, it is necessary to first determine the corresponding field test method.

Major difficulties still arise when testing drone spray in wind tunnels. The principle of drift testing in wind tunnel is that it should have a sufficient size so that the airflow is not disturbed by the inner wall or sprayer (or its installation) (Iso 22856, 2008). Moreover, height and downwind distance of the wind tunnel should be sufficient to contain enough sampling equipment or collectors. According to Table 3, the length of the UASS is generally greater than 2 m, the height range lies between 0.5 and 0.7 m, and the size range of the rotor is 0.53–1.19 m. According to the specifications of the wind tunnel for spray testing mentioned in the literature (Ling et al., 2018; Ding et al., 2019; Liao et al., 2019; Wang et al., 2020, 2020b), the width of most wind tunnel cross-sections ranges between 1.2 and 3 m, while the height is 1.1–2 m high. As the length of the rotor itself interferes with the current cross-sectional dimensions of the wind tunnel, the whole machine or the rotor are difficult to place inside the wind tunnel. The test method of UASS drift should take into account the characteristics of the UASS system. For example, the rotor wind field may impact the movement of the droplets as well as the secondary atomization. However, the ISO 22856 standard mentions that the spray generator mounting, control, and supply lines are to be arranged in order to minimize disturbance to the airflow, thus leading to a contradiction. It is therefore crucial to revise the field and indoor drift test methods based on the characteristics of UASS spraying systems.

## Conclusion

The rapid development of drone sprayers has provided novel opportunities for chemical spraying techniques, but the drift of the UASS is also a noteworthy feature. The high-speed motion of the rotor causes the droplets to be drawn in by vortices on both sides of the wingtips, while the forward motion of the aircraft causes the vortices to produce long trailing vortices at the rear. Under the combined action of the lateral wind and the wake vortex, the droplets are easily dispersed toward the non-target areas. However, drone drifting is not an uncontrollable phenomenon. UASS drift has been found to be affected by the droplet size, layout of nozzles, number and size of rotors, payload, flying speed, flying altitude, and crosswind. By optimizing the structural layout of the rotor and spraying system, adjusting the operating parameters, and establishing a drift buffer zone, the drift of the droplets can be effectively reduced. For this new spray equipment, it is necessary for researchers to further investigate the drift characteristics of UASS, establish drift models of typical models, crops, and climate environment, and discuss standard methods for measuring UASS drift.

## Author contributions

JD, YL, and PC: conceptualization. PC: formal analysis and writing—original draft preparation. PC, JD, EC, XD, and GP: investigation. JD and YL: resources, supervision, and funding acquisition. JD, YL, EC, XD, and GP: writing—review and editing. YZ: visualization. All authors have read and agreed to the published version of the manuscript.

## References

[B1] Ahmad F. Zhang S. Qiu B. Ma J. Xin H. Qiu W. (2022). Comparison of water sensitive paper and glass strip sampling approaches to access spray deposit by UAV sprayers. *Agronomy* 12:1302. 10.3390/agronomy12061302

[B2] Al Heidary M. Douzals J. P. Sinfort C. Vallet A. (2014). Influence of spray characteristics on potential spray drift of field crop sprayers: a literature review. *Crop Prot.* 63 120–130. 10.1016/j.cropro.2014.05.006

[B3] ASAE ANSI/ASABE (2020). *Spray nozzle classification by droplet spectra. Standard 572.3*. St. Joseph, MI: American Society of Agricultural and Biological Engineers.

[B4] Balsari P. Grella M. Marucco P. Matta F. Miranda Fuentes A. (2019). Assessing the influence of air speed and liquid flow rate on the droplet size and homogeneity in pneumatic spraying. *Pest. Manag. Sci.* 75 366–379. 10.1002/ps.5120 29920925

[B5] Bartzanas T. Kacira M. Zhu H. Karmakar S. Tamimi E. Katsoulas N. (2013). Computational fluid dynamics applications to improve crop production systems. *Comput. Electron. Agric.* 93 151–167. 10.1016/j.compag.2012.05.012

[B6] Bloise N. Ruiz M. C. D’Ambrosio D. Guglieri G. (2020). “Preliminary design of a remotely piloted aircraft system for crop-spraying on vineyards,” in *Proceedings of the 2020 IEEE International Workshop on Metrology for Agriculture and Forestry (MetroAgriFor).* (Trento: IEEE), 1–6. 10.1109/MetroAgriFor50201.2020.9277607

[B7] Brown C. R. Giles D. K. (2018). Measurement of pesticide drift from unmanned aerial vehicle application to a vineyard. *Transac. Asabe* 61 1539–1546. 10.13031/trans.12672

[B8] Butler Ellis M. C. Swan T. Miller P. Waddelow S. Bradley A. Tuck C. R. (2002). PM—power and machinery: design factors affecting spray characteristics and drift performance of air induction nozzles. *Biosyst. Eng.* 82 289–296. 10.1006/bioe.2002.0069

[B9] Cavalaris C. Karamoutis C. Markinos A. (2022). Efficacy of cotton harvest aids applications with unmanned aerial vehicles (UAV) and ground-based field sprayers–a case study comparison. *Smart Agric. Technol.* 2:100047. 10.1016/j.atech.2022.100047

[B10] Chen H. Lan Y. Fritz B. K. Hoffmann W. C. Liu S. (2021). Review of agricultural spraying technologies for plant protection using unmanned aerial vehicle (UAV). *Int. J. Agric. Biol. Eng.* 14 38–49. 10.25165/j.ijabe.20211401.5714

[B11] Chen P. Lan Y. Douzals J. Ouyang F. Wang J. Xu W. (2020a). Droplet distribution of Unmanned Aerial Vehicle under several spray volumes and canopy heights in the cotton canopy. *Int. J. Precis. Agric. Aviat.* 3 74–79. 10.33440/j.ijpaa.20200304.136

[B12] Chen P. Lan Y. Huang X. Qi H. Wang G. Wang J. (2020b). Droplet deposition and control of planthoppers of different nozzles in two-stage rice with a quadrotor unmanned aerial vehicle. *Agronomy* 10:303. 10.3390/agronomy10020303

[B13] Chen P. Ouyang F. Wang G. Qi H. Xu W. Yang W. (2021). Droplet distributions in cotton harvest aid applications vary with the interactions among the unmanned aerial vehicle spraying parameters. *Ind. Crop. Prod.* 163:113324. 10.1016/j.indcrop.2021.113324

[B14] Chen P. Xu W. Zhan Y. Wang G. Yang W. Lan Y. (2022). Determining application volume of unmanned aerial spraying systems for cotton defoliation using remote sensing images. *Comput. Electron. Agric.* 196:106912. 10.1016/j.compag.2022.106912

[B15] Chen S. Lan Y. Zhou Z. Deng X. Wang J. (2021). Research advances of the drift reducing technologies in application of agricultural aviation spraying. *Int. J. Agric. Biol. Eng.* 14 1–10. 10.25165/j.ijabe.20211405.6225

[B16] Chen S. Lan Y. Zhou Z. Ouyang F. Wang G. Huang X. (2020a). Effect of droplet size parameters on droplet deposition and drift of aerial spraying by using plant protection UAV. *Agronomy* 10:195. 10.3390/agronomy10020195

[B17] Delpuech X. Gorioux H. Pouxviel G. (2022). *Évaluation de la Qualité de la Pulvérisation par Drone en Vignoble de forte pente: Article Prenant sa Source de l’article “Pulvérisation par Drone en vignoble de forte pente”(Phytoma-La santé des végétaux n^°^ 741, février 2021).* Paris: vine and wine. 10.20870/IVES-TR.2022.5402

[B18] Ding S. Xue X. Qin W. Gu W. Cai C. Cui L. (2019). Influencing factors research and performance experiment on droplets deposition at low wind speed. *Int. J. Precis. Agric. Aviat.* 2 46–51. 10.33440/j.ijpaa.20190201.0017

[B19] Fengbo Y. Xinyu X. Ling Z. Zhu S. (2017). Numerical simulation and experimental verification on downwash air flow of six-rotor agricultural unmanned aerial vehicle in hover. *Int. J. Agric. Biol. Eng.* 10 41–53. 10.25165/j.ijabe.20171004.3077

[B20] Ferguson J. C. O’Donnell C. C. Chauhan B. S. Adkins S. W. Kruger G. R. Wang R. (2015). Determining the uniformity and consistency of droplet size across spray drift reducing nozzles in a wind tunnel. *Crop Prot.* 76 1–6. 10.1016/j.cropro.2015.06.008

[B21] Gao Y. Y. (2013). *Study on Distribution of Pesticide Droplets in Gramineous Crop Canopy And Control Effect Sprayed By Unmanned Aerial Vehicle.* Harbin: Northeast Agric. Univ.

[B22] Grella M. Marucco P. Manzone M. Gallart M. Balsari P. (2017). Effect of sprayer settings on spray drift during pesticide application in poplar plantations (*Populus* spp.). *Sci. Total Environ*. 578, 427–439. 10.1016/j.scitotenv.2016.10.205 27836339

[B23] Hayashi H. Takeda S. (1986). Spray drying characteristics by a centrifugal pressure nozzle with large orifice diameter. *Dry. Technol.* 4 331–342. 10.1080/07373938608916333

[B24] He X. (2018). Rapid development of unmanned aerial vehicles (UAV) for plant protection and application technology in China. *Outlooks Pest Manag.* 29 162–167. 10.1564/v29_aug_04

[B25] He X. Bonds J. Herbst A. Langenakens J. (2017). Recent development of unmanned aerial vehicle for plant protection in East Asia. *Int. J. Agric. Biol. Eng.* 10 18–30. 10.3965/j.ijabe.20171003.3248

[B26] He Y. Xiao S. Fang H. Dong T. Tang Y. Nie P. (2018). Development situation and spraying decision of spray nozzle for plant protection UAV. *Transac. Chin. Soc. Agric. Eng.* 34 113–124. 10.11975/j.issn.1002-6819.2018.13.014

[B27] Herbst A. Bonds J. Wang Z. C. Zeng A. J. He X. K. Goff P. (2020). The influence of unmanned agricultural aircraft system design on spray drift. *J. Kulturpfl.* 72 1–11. 10.5073/JfK.2020.01.01

[B28] Hong S. Park J. Jeong H. Lee S. Choi L. Zhao L. (2021). Fluid dynamic approaches for prediction of spray drift from ground pesticide applications: A review. *Agronomy* 11:1182. 10.3390/agronomy/11061182

[B29] Hussain A. Nishat A. S. (2022). *The Energy Challenge: Moving from Fossil Fuels to Biofuels, Hydrogen, and Green Energy Sources.* New York, NY: New York City College of Technology.

[B30] Ismail S. A. Yahya A. Su A. S. M. Asib N. Mustafah A. M. (2021). Drone payload and flying speed effects on rotor blades’ RPM and traveling pattern for agricultural chemical spraying. *Basrah J. Agric. Sci*. 34, 157–170. 10.37077/25200860.2021.34.sp1.16

[B31] Iso I. (2005). *Equipment for Crop Protection-Methods for field Measurements of Spray Drift.* Geneva: International Organization for Standardization, 1–17.

[B32] Iso International Standard (2009). *Equipment for Crop Protection—Methods for the Laboratory Measurement of spray Drift—Wind Tunnels.* Geneva: ISO.

[B33] Lan Y. Chen S. (2018). Current status and trends of plant protection UAV and its spraying technology in China. *Int. J. Precis. Agric. Aviat.* 1:1. 10.33440/j.ijpaa.20180101.0002

[B34] Li J. Lan Y. Shi Y. (2018). Research progress on airflow characteristics and field pesticide application system of rotary-wing UAV. *Transac. Chin. Soc. Agric. Eng.* 34 104–118. 10.3969/j.issn.1002-6819.2018.12.013

[B35] Li L. Liu Y. He X. Song J. Zeng A. Zhichong W. (2018). *Assessment of Spray Deposition and Losses in the Apple Orchard from Agricultural Unmanned Aerial Vehicle in China.* St. Joseph, MI: American Society of Agricultural and Biological Engineers. 10.13031/aim.201800504

[B36] Li X. Giles D. K. Andaloro J. T. Long R. Lang E. B. Watson L. J. (2021a). Comparison of UAV and fixed-wing aerial application for alfalfa insect pest control: evaluating efficacy, residues, and spray quality. *Pest Manag. Sci.* 77 4980–4992. 10.1002/ps.6540 34216079

[B37] Li X. Giles D. K. Niederholzer F. J. Andaloro J. T. Lang E. B. Watson L. J. (2021b). Evaluation of an unmanned aerial vehicle as a new method of pesticide application for almond crop protection. *Pest Manage. Sci.* 77 527–537. 10.1002/ps.6052 32816397

[B38] Liao J. Hewitt A. J. Wang P. Luo X. Zang Y. Zhou Z. (2019). Development of droplet characteristics prediction models for air induction nozzles based on wind tunnel tests. *Int. J. Agric. Biol. Eng.* 12 1–6. 10.25165/j.ijabe.20191206.5014

[B39] Ling W. Du C. Ze Y. Xindong N. Shumao W. (2018). Research on the prediction model and its influencing factors of droplet deposition area in the wind tunnel environment based on UAV spraying. *IFAC PapersOnLine* 51 274–279. 10.1016/j.ifacol.2018.08.174

[B40] Liu Q. Chen S. Wang G. Lan Y. (2021). Drift evaluation of a quadrotor unmanned aerial vehicle (UAV) sprayer: effect of liquid pressure and wind speed on drift potential based on wind tunnel test. *Appl. Sci.* 11:7258. 10.3390/app11167258

[B41] Meng Y. Lan Y. Mei G. Guo Y. Song J. Wang Z. G. (2018). Effect of aerial spray adjuvant applying on the efficiency of small unmanned aerial vehicle for wheat aphids control. *Int. J. Agric. Biol. Eng.* 11 46–53. 10.25165/j.ijabe.20181105.4298

[B42] Meng Y. Su J. Song J. Chen W. Lan Y. (2020). Experimental evaluation of UAV spraying for peach trees of different shapes: effects of operational parameters on droplet distribution. *Comput. Electron. Agric.* 170:105282. 10.1016/j.compag.2020.105282

[B43] Meng Y. Wang M. Wang Z. Hu H. Ma Y. (2021). Surface tension and spreading coefficient of single-and mix-pesticide solutions with aerial spraying organosilicone adjuvant. *Int. J. Precis. Agric. Aviat.* 4 6–13. 10.33440/j.ijpaa.20210401.159

[B44] Meng Y. Zhong W. Liu C. Su J. Su J. Lan Y. (2022a). UAV spraying on citrus crop: impact of tank-mix adjuvant on the contact angle and droplet distribution. *PeerJ* 10:e13064. 10.7717/peerj.13064 35295557 PMC8919849

[B45] Meng Y. Zhong W. Liu Y. Wang M. Lan Y. (2022b). *Droplet Distribution of an Autonomous UAV-Based Sprayer in Citrus Tree Canopy.* Bristol: IOP Publishing. 10.1088/1742-6596/2203/1/012022

[B46] Mickle R. E. (1996). Influence of aircraft vortices on spray cloud behavior. *J. Am. Mosq. Control Assoc. Mosq. News* 12 372–379. 8827623

[B47] Morales-Rodríguez P. A. Cano Cano E. Villena J. López-Perales J. A. (2022). A comparison between conventional sprayers and new UAV sprayers: A study case of vineyards and olives in extremadura (Spain). *Agronomy* 12:1307. 10.3390/agronomy12061307

[B48] Pan Z. Lie D. Qiang L. Shaolan H. Shilai Y. Yande L. (2016). Effects of citrus tree-shape and spraying height of small unmanned aerial vehicle on droplet distribution. *Int. J. Agric. Biol. Eng.* 9:45. 10.3965/j.ijabe.20160904.2178

[B49] Qin W. Qiu B. Xue X. Chen C. Xu Z. Zhou Q. (2016). Droplet deposition and control effect of insecticides sprayed with an unmanned aerial vehicle against plant hoppers. *Crop Prot.* 85 79–88. 10.1016/j.cropro.2016.03.018

[B50] Qingqing Z. Xinyu X. Weicai Q. Chen C. Liangfu Z. (2017). Optimization and test for structural parameters of UAV spraying rotary cup atomizer. *Int. J. Agric. Biol. Eng.* 10 78–86. 10.3965/j.ijabe.20171003.3119

[B51] Reger M. Bauerdick J. Bernhardt H. (2018). Drones in agriculture: current and future legal status in Germany, the EU, the USA and Japan. *Landtechnik* 73 62–79. 10.15150/lt.2018.3183

[B52] Richardson B. Rolando C. A. Kimberley M. O. Strand T. M. (2019). Spray application efficiency from a multi-rotor unmanned aerial vehicle configured for aerial pesticide application. *Trans. Asabe* 62 1447–1453. 10.13031/trans.13509 31595645

[B53] Sarri D. Martelloni L. Rimediotti M. Lisci R. Lombardo S. Vieri M. (2019). Testing a multi-rotor unmanned aerial vehicle for spray application in high slope terraced vineyard. *J. Agric. Eng.* 50 38–47. 10.4081/jae.2019.853

[B54] Shan C. Wang G. Wang H. Xie Y. Wang H. Wang S. (2021). Effects of droplet size and spray volume parameters on droplet deposition of wheat herbicide application by using UAV. *Int. J. Agric. Biol. Eng.* 14 74–81. 10.25165/j.ijabe.20211401.6129

[B55] Shi Q. Mao H. Guan X. (2019). Numerical simulation and experimental verification of the deposition concentration of an unmanned aerial vehicle. *Appl. Eng. Agric.* 35 367–376. 10.13031/aea.13221

[B56] Song C. Zhou Z. Jiang R. Luo X. He X. Ming R. (2018). Design and parameter optimization of pneumatic rice sowing device for unmanned aerial vehicle. *Transac. Chin. Soc. Agric. Eng.* 34 80–88. 10.11975/j.issn.1002-6819.2018.06.010

[B57] Tang Q. Chen L. Zhang R. Deng W. Xu M. Xu G. (2021). Effects of application height and crosswind on the crop spraying performance of unmanned helicopters. *Comput. Electron. Agric.* 181:105961. 10.1016/j.compag.2020.105961

[B58] Tang Q. Zhang R. Chen L. Xu G. Deng W. Ding C. (2020). High-accuracy, high-resolution downwash flow field measurements of an unmanned helicopter for precision agriculture. *Comput. Electron. Agric.* 173:105390. 10.1016/j.compag.2020.105390

[B59] Teske M. E. Wachspress D. A. Thistle H. W. (2018). Prediction of aerial spray release from UAVs. *Transac. Asabe* 61 909–918. 10.13031/trans.12701

[B60] Wang C. He X. Wang X. Wang Z. Pan H. He Z. (2016). Testing method of spatial pesticide spraying deposition quality balance for unmanned aerial vehicle. *Transac. Chin. Soc. Agric. Eng.* 32 54–61. 10.25165/j.ijabe.20181102.3187

[B61] Wang C. He X. Wang X. Wang Z. Wang S. Li L. (2018). Testing method and distribution characteristics of spatial pesticide spraying deposition quality balance for unmanned aerial vehicle. *Int. J. Agric. Biol. Eng.* 11 18–26.

[B62] Wang C. He X. Zeng A. Herbst A. Wanlin G. (2020a). Measuring method and experiment on spray drift of chemicals applied by uav sprayer based on an artificial orchard test bench. *Transac. Chin. Soc. Agric. Eng.* 36 56–66. 10.11975/j.issn.1002-6819.2020.13.007

[B63] Wang C. Zeng A. He X. Song J. Herbst A. Gao W. (2020b). Spray drift characteristics test of unmanned aerial vehicle spray unit under wind tunnel conditions. *Int. J. Agric. Biol. Eng.* 13 13–21. 10.25165/j.ijabe.20201303.5716

[B64] Wang C. Herbst A. Zeng A. Wongsuk S. Qiao B. Qi P. (2021). Assessment of spray deposition, drift and mass balance from unmanned aerial vehicle sprayer using an artificial vineyard. *Sci. Total Environ.* 777:146181. 10.1016/j.scitotenv.2021.146181 33689892

[B65] Wang C. Liu Y. Zhang Z. Han L. Li Y. Zhang H. (2022). Spray performance evaluation of a six-rotor unmanned aerial vehicle sprayer for pesticide application using an orchard operation mode in apple orchards. *Pest Manag. Sci.* 78 2449–2466. 10.1002/ps.6875 35306733

[B66] Wang G. Han Y. Li X. Andaloro J. Chen P. Hoffmann W. C. (2020). Field evaluation of spray drift and environmental impact using an agricultural unmanned aerial vehicle (UAV) sprayer. *Sci. Total Environ.* 737:139793. 10.1016/j.scitotenv.2020.139793 32526578

[B67] Wang G. Lan Y. Yuan H. Qi H. Chen P. Ouyang F. (2019). Comparison of spray deposition, control efficacy on wheat aphids and working efficiency in the wheat field of the unmanned aerial vehicle with boom sprayer and two conventional knapsack sprayers. *Appl. Sci.* 9. 10.3390/app9020218

[B68] Wang J. Lan Y. Wen S. Hewitt A. J. Yao W. Chen P. (2019a). Meteorological and flight altitude effects on deposition, penetration, and drift in pineapple aerial spraying. *Asia Pac. J. Chem. Eng.* 15. 10.1002/apj.2382

[B69] Wang J. Lan Y. Yao W. Chen P. Lin J. Yan Y. (2019b). Effects of working height of single-rotor unmanned aerial vehicle on drift and droplets deposition distribution of areca tree. *Nongye Jixie Xuebao* 50 10.6041/j.issn.1000-1298.2019.07.011

[B70] Wang J. Lan Y. Zhang H. Zhang Y. Wen S. Yao W. (2018). Drift and deposition of pesticide applied by UAV on pineapple plants under different meteorological conditions. *Int. J. Agric. Biol. Eng.* 11 5–12. 10.25165/j.ijabe.20181106.4038

[B71] Wang J. Xu W. Wen J. Wang X. Luo B. (2017). Numerical simulation on gas-liquid phase flow of large-scale plant protection unmanned aerial vehicle spraying. *Transac. Chin. Soc. Agric. Mach.* 48.

[B72] Wang L. Huang X. Li W. Yan K. Han Y. Zhang Y. (2022). Progress in agricultural unmanned aerial vehicles (UAVs) applied in China and prospects for Poland. *Agriculture* 12:397. 10.3390/agriculture12030397

[B73] Wang L. Lan Y. Zhang Y. Zhang H. Tahir M. N. Ou S. (2019). Applications and prospects of agricultural unmanned aerial vehicle obstacle avoidance technology in China. *Sensors* 19:642. 10.3390/s19030642 30717488 PMC6387432

[B74] Wang X. He X. Song J. Wang Z. Wang C. Wang S. (2018). Drift potential of UAV with adjuvants in aerial applications. *Int. J. Agric. Biol. Eng.* 11 54–58. 10.25165/j.ijabe.20181105.3185

[B75] Wang X. He X. Wang C. Wang Z. Li L. Wang S. (2017). Spray drift characteristics of fuel powered single-rotor UAV for plant protection. *Transac. Chin. Soc. Agric. Eng.* 33 117–123. 10.11975/j.issn.1002-6819.2017.01.016

[B76] Wang Z. Hussain M. Huang G. Yin J. Guo Y. Mo Y. (2022). Better droplet deposition and internode shortening effects of plant growth regulator EDAH on maize applied by small unmanned aerial vehicle than electric knapsack sprayer. *Agriculture* 12:404. 10.3390/agriculture12030404

[B77] Wen S. Han J. Lan Y. B. Yin X. C. Lu Y. H. (2018). Influence of wing tip vortex on drift of single rotor plant protection unmanned aerial vehicle. *Trans. Chin. Soc. Agric. Mach.* 49:127â. 10.6041/j.issn.1000-1298.2018.08.015

[B78] Wen S. Han J. Ning Z. Lan Y. Yin X. Zhang J. (2019). Numerical analysis and validation of spray distributions disturbed by quad-rotor drone wake at different flight speeds. *Comput. Electron. Agric*. 166:105036. 10.1016/j.compag.2019.105036

[B79] Xinyu X. Kang T. Weicai Q. Lan Y. Zhang H. (2014). Drift and deposition of ultra-low altitude and low volume application in paddy field. *Int. J. Agric. Biol. Eng.* 7:23. 10.3965/j.ijabe.20140704.003

[B80] Xu S. Jianli S. Shilin W. Xiaoming J. Lin X. Yajia L. (2020). Study on droplet drift and applicator exposure in rice flight prevention by multi-rotor plant protection UAV. *Chin. J. Pest. Sci.* 22 1085–1093. 10.16801/j.issn.1008-7303.2020.0148

[B81] Yan X. Xin S. Xiaohui L. Du Yahui Daibin Y. Huizhu Y. (2021). The spray drift risk of plant protection unmanned aerial vehicle (UAV spraying neonicotinoid pesticides to honey bees. *J. Plant Prot.* 48 477–482. 10.13802/j.cnki.zwbhxb.2021.2021836

[B82] Yan X. Yuan H. Chen Y. Shi X. Liu X. Wang Z. (2022). Broadcasting of tiny granules by drone to mimic liquid spraying for the control of fall armyworm (*Spodoptera frugiperda*). *Pest Manag. Sci.* 78 43–51. 10.1002/ps.6604 34405509

[B83] Yu K. Liu Y. Gong Z. Liang Y. Du L. Zhang Z. (2022). Chemical topping improves the efficiency of spraying harvest aids using unmanned aerial vehicles in high-density cotton. *Field Crops Res.* 283:108546. 10.1016/j.fcr.2022.108546

[B84] Yuan H. Z. Xue X. Y. Yan X. J. Qin W. C. Kong X. Zhou Y. Y. (2018). Applications and prospects in the unmanned aerial system for low-altitude and low-volume spray in crop protection. *Plant Protect.* 44 152–158. 10.16688/j.zwbh.2018307

[B85] Zhai C. Zhao C. Ning W. Long J. Wang X. Weckler P. (2018). Research progress on precision control methods of air-assisted spraying in orchards. *Transac. Chin. Soc. Agric. Eng.* 34 1–15. 10.11975/j.issn.1002-6819.2018.10.001

[B86] Zhan Y. Chen P. Xu W. Chen S. Han Y. Lan Y. (2022). Influence of the downwash airflow distribution characteristics of a plant protection UAV on spray deposit distribution. *Biosyst. Eng.* 216 32–45. 10.1016/j.biosystemseng.2022.01.016

[B87] Zhang H. Qi L. Wan J. Musiu E. M. Zhou J. Lu Z. (2022). Numerical simulation of downwash airflow distribution inside tree canopies of an apple orchard from a multirotor unmanned aerial vehicle (UAV) sprayer. *Comput. Electron. Agric.* 195:106817. 10.1016/j.compag.2022.106817

[B88] Zhang H. Qi L. Wu Y. Musiu E. M. Cheng Z. Wang P. (2020). Numerical simulation of airflow field from a six–rotor plant protection drone using lattice Boltzmann method. *Biosyst. Eng.* 197 336–351. 10.1016/j.biosystemseng.2020.07.018

[B89] Zhang S. Qiu B. Xue X. Sun T. Peng B. (2020). Parameters optimization of crop protection UAS based on the first industry standard of China. *Int. J. Agric. Biol. Eng.* 13 29–35. 10.25165/j.ijabe.20201303.5439

[B90] Zhang Y. Huang X. Lan Y. Wang L. Lu X. Yan K. (2021). Development and prospect of UAV-based aerial electrostatic spray technology in China. *Appl. Sci.* 11:4071. 10.3390/app11094071

[B91] Zhu H. Li H. Zhang C. Li J. Zhang H. (2019). Performance characterization of the UAV chemical application based on CFD simulation. *Agronomy* 9:308. 10.3390/agronomy9060308

