# Supplementary material for: Characteristics of unmanned aerial spraying systems and related spray drift: A review
Source: Front Plant Sci. 2022 Aug 8;13:870956. doi: 10.3389/fpls.2022.870956 (PMC9395147; doi:10.3389/fpls.2022.870956)
Supplement: Supplementary file 1 [file Data_Sheet_1.PDF]

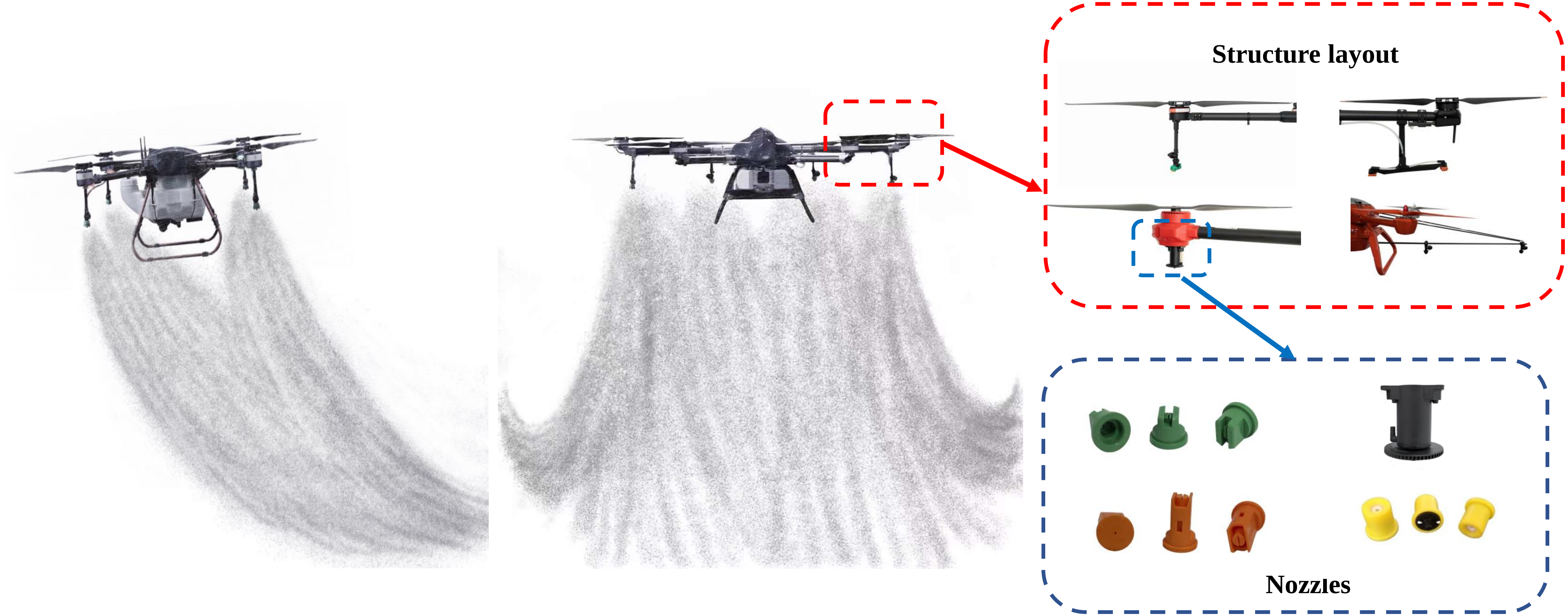

Forward movement **Downwash airflow (hover state)** **Spray system and Structure layout**

The example UASS is Liberty Eagle TP-32, Source by Quanfeng Aviation, Anyang, China
